# The Need for Monopolising the Milk Supply

**Published:** 1908-11-14

**Authors:** 


### Public Health and Hygiene.

### THE NEED FOR MONOPOLISING THE MILK SUPPLY.

We have repeatedly referred to the hygienic anomalies which beset the milk supply of this country. From cow to consumer the history of average milk is one long series of disgusting and dangerous pollutions. Every medical officer of health knows that existing administrative supervision utterly fails to secure anything approaching a hygienic supply. Perforce, and as a makeshift, he has urged that milk should be boiled or pasteurised as some measure of protection against the foul iniquities of milk as it is. He is only too conscious that the machinery at his disposal fails, and must fail, to secure clean milk. Every inspection discovers some actual or potential fouling; and it is like trying to sweep back the tide, to deal with the particular instances which come before him, while the ocean of ignorant incompetence in the trade surges around him. It is not that there are not enlightened and intelligent milk vendors, zealous for the good fame and quality of their milk, who take a proper pride in their efforts to supply an honest and untainted article. They vie with the officers of the health service in their desire to protect the commodity in which they are so intimately concerned; but, like the health officers, they are powerless to do more than leaven in some small degree the lump supply.

The officers of the public health service cannot be ubiquitous. They have many duties to perform other than supervising the milk supply; and properly to supervise and reform this trade, so as to ensure reasonably and decently clean milk, would more than absorb all their time. It is only when the milk service is fully organised, and is inspired by a conscience sensitive to the dangers of microscopical dirt, as well as of gross filth, that there can be any hope of a general milk supply above suspicion.

A recent visit paid to a dairy of the Copenhagen Milk Supply Company has shown us the marked contrast in which a well-organised trade can secure the cleanliness of its milk as compared with the usual conditions under which it is supplied in this country.

The farms from which the Copenhagen Company draws its milk are under systematic bi-monthly inspections by veterinary surgeons. The cows are groomed and the udders washed before milking. The milk is received into ice-chilled pails, is rapidly cooled and filtered. The churns in which it is transmitted to the central depots are sealed at the farm with leaden seals, so that there is no possibility of the milk being polluted in transit. It is conveyed in specially constructed vans, in which the churns are packed in ice. Arrived at the depot, the churns are taken direct from the railway van within the company's premises to the distributing room, and there, in the presence of a responsible official of the company, the seal is broken, the source and time of milking noted, the milk sampled and tasted, its temperature taken, and the sample from each churn sent to the laboratory. The milk is now passed through a clean, carefully prepared gravel filter, from which it is withdrawn in taps into clean bottles, and forthwith corked, sealed, and labelled. The bottles are then packed in ice in wooden cases and distributed, each with its leaden seal unbroken, to the consumer.

The arrangements in the dairy for securing the cleanliness of the vessels and of the employees leave nothing which careful and zealous forethought could suggest. The necessary manipulations in the distribution of the milk are ingeniously devised, so that there is literally no handling of the milk at any stage until it reaches the consumer. The system of sealing, sampling, and labelling enables each consignment of milk to be traced right back to its source, as well as to be intercepted at the depot should its examination reveal any defect in quality or physical condition. Cooled immediately after leaving the cow, it is kept at a low temperature until delivered to the consumer. The aim of the company, in a word, is clean unsophisticated milk, and, so far as we could judge, this aim is attained.

Contrast with this the condition of the average London milk! In large part that comes from cow-sheds which are the despair of the sanitarian. The cow-keeper, as a rule, is so little perturbed by the admixture of cow-dung inseparable from his methods of milking, that not improbably, if remonstrated with, he will express an opinion on the advantages of cow-dung as an article of diet. We have known it claimed under such circumstances as a prophylactic against tuberculosis. From the cow-shed the milk is conveyed in cans which at any stage of the journey can be opened by authorised or unauthorised persons; they are so constructed that they fail to prevent the admission of dirt to the milk even if unopened. The railway vans in which they are conveyed bear no sort of structural relation to the purpose for which they are used. No attempt is made to keep the milk cool in transit; no effort at supervision at the different stages of its journey to the milk vendor. Frequently the origin of any particular sample is untraceable, as the milk from several farms may all be mixed together. In the retail shops it is not uncommonly kept in open vessels on the counter or shop floor, and there it becomes the medium of extinction of innumerable flies and a receptacle for clouds of floor sweepings. All these things and worse can be seen by anyone in the general shops where milk is sold.

The only plan for effectively removing these disgusting anomalies is to treat the milk supply as the water supply of large towns has been treated. Whether the monopoly should be vested in an authorised company, a local authority, or a specially constituted authority is a larger question. As sanitarians, it is enough that we insist upon the only practical solution of an important hygienic problem. With an organised control over the whole milk trade of a locality, inspired from within by the ideals of the sanitarian, there would be an economy in cost of distribution and a vast improvement in purity and quality of what, after all, is more a prime necessity of life than either coal, gas, or electricity.

